# Task-discriminative space-by-time factorization of muscle activity

**DOI:** 10.3389/fnhum.2015.00399

**Published:** 2015-07-10

**Authors:** Ioannis Delis, Stefano Panzeri, Thierry Pozzo, Bastien Berret

**Affiliations:** ^1^Institute of Neuroscience and Psychology, University of GlasgowGlasgow, UK; ^2^Neural Computation Laboratory, Center for Neuroscience and Cognitive Systems@UniTn, Istituto Italiano di TecnologiaRovereto, Italy; ^3^Robotics, Brain and Cognitive Sciences Department, Istituto Italiano di TecnologiaGenoa, Italy; ^4^Institut Universitaire de France, Université de BourgogneDijon, France; ^5^INSERM, U1093, Cognition Action Plasticité SensorimotriceDijon, France; ^6^CIAMS, EA 4532, Univ Paris-SudOrsay, France

**Keywords:** muscle synergies, task space, primitives, modularity, dimensionality reduction

## Abstract

Movement generation has been hypothesized to rely on a modular organization of muscle activity. Crucial to this hypothesis is the ability to perform reliably a variety of motor tasks by recruiting a limited set of modules and combining them in a task-dependent manner. Thus far, existing algorithms that extract putative modules of muscle activations, such as Non-negative Matrix Factorization (NMF), identify modular decompositions that maximize the reconstruction of the recorded EMG data. Typically, the functional role of the decompositions, i.e., task accomplishment, is only assessed *a posteriori*. However, as motor actions are defined in task space, we suggest that motor modules should be computed in task space too. In this study, we propose a new module extraction algorithm, named DsNM3F, that uses task information during the module identification process. DsNM3F extends our previous space-by-time decomposition method (the so-called sNM3F algorithm, which could assess task performance only after having computed modules) to identify modules gauging between two complementary objectives: reconstruction of the original data and reliable discrimination of the performed tasks. We show that DsNM3F recovers the task dependence of module activations more accurately than sNM3F. We also apply it to electromyographic signals recorded during performance of a variety of arm pointing tasks and identify spatial and temporal modules of muscle activity that are highly consistent with previous studies. DsNM3F achieves perfect task categorization without significant loss in data approximation when task information is available and generalizes as well as sNM3F when applied to new data. These findings suggest that the space-by-time decomposition of muscle activity finds robust task-discriminating modular representations of muscle activity and that the insertion of task discrimination objectives is useful for describing the task modulation of module recruitment.

## 1. Introduction

The hypothesis of modularity in muscle activity relies on the premise that the central nervous system (CNS) stores a limited set of modules and recruits them to execute the motor tasks at hand (Bizzi et al., 2008; Tresch and Jarc, 2009; Bizzi and Cheung, 2013). Putative modules are typically identified by means of dimensionality reduction algorithms applied to electromyographic (EMG) recordings (Tresch et al., 2006). This procedure yields modular decompositions that approximate the EMG data as accurately as possible despite the significant measurement noise that affects such data. Different types of modularity have been proposed assuming the existence of either spatial (Tresch et al., 1999; Ting and Macpherson, 2005), temporal (Ivanenko et al., 2004, 2005), or spatiotemporal modules (d'Avella et al., 2003; Russo et al., 2014). Recently, we developed a unifying modularity model, termed as space-by-time decomposition, which encompasses and merges both space and time dimensions and provides a low-dimensional yet accurate, highly flexible and task-relevant representation of muscle patterns (Delis et al., 2014). To implement it, we introduced a specific dimensionality reduction algorithm based on non-negative matrix factorization (NMF). This sample-based non-negative matrix tri-factorization (sNM3F) identifies concurrent spatial and temporal modules and combines them with scalar coefficients in order to reconstruct the single-sample EMG signals. To assess the functionality of this representation, we tested it in task space *a posteriori*, i.e., we evaluated the effectiveness of the decomposition in categorizing correctly the task performed in each single sample. We formalized this assessment in terms of a task-decoding metric (Delis et al., 2013a). This methodology comprised two independent steps: (a) extraction of spatial and temporal modules with the aim of approximating the EMG data as accurately as possible without regard to task discrimination and (b) evaluation of the performance of a decomposition using a task-decoding metric and choosing the smallest set of modules that give the highest task discrimination performance.

However, as motor actions are defined in task space, we suggest that motor modules should be computed in task space too (Alessandro et al., 2013). In this study, we propose a method that we consider as a first step in the direction of task-space module identification. Ultimately, such an approach is crucial for the use of modular decompositions in practical and clinical applications, such as body-machine interfaces, control of robotic limbs, and rehabilitation devices.

**Figure 1:**
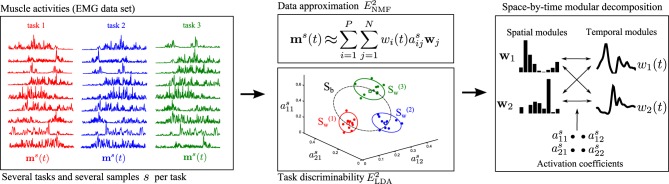
**Schematic illustration of the steps of the proposed module identification approach**. **Left:** The activity of several muscles is recorded simultaneously during performance of several repetitions of a variety of motor tasks. For illustration, here we show the activation of nine muscles when performing three tasks (task1 in red, task2 in blue, task3 in green). **Middle and Top:** The first objective of DsNM3F is to approximate the muscle activations by a lower-dimensional representation defined by *P* temporal modules *w*_*i*_(*t*) and *N* spatial modules ***w*_*j*_**. *E*^2^_*NMF*_ is the squared residual error in this approximation, which is minimized by the algorithm and is standard in dimensionality reduction methods. **Middle and Bottom:** The second objective of DsNM3F is to categorize the performed tasks as unequivocally as possible. This is achieved in the space defined by module activations *a*^*s*^. Open green, blue, and red dots represent single-sample module activations to perform task1, task2, and task3, respectively and squares represent the means of activation for each task. For visualization, we show three activation coefficients here but this space is in general of dimension *P* × *N*. To discriminate between tasks, the dispersion of module activations within each task (*S*^(1)^_*w*_, *S*^(2)^_*w*_,*S*^(3)^_*w*_ shown as red, blue, and green ellipses, respectively) should be minimized, whereas the dispersion of module activations across tasks (*S*_*b*_ shown as a dashed-line black ellipse) should be maximized. *E*^2^_*LDA*_ is the task discrimination cost expressed as a linear combination of *S*_*w*_and *S*_*b*_. **Right:** The proposed method integrates the data approximation and the task discrimination objective to identify invariant spatial/temporal modules and activation coefficients that combine them in single samples. For illustration, we show two spatial and two temporal modules combined with 2×2=4 activation coefficients to reconstruct each sample of muscle activity.

Our proposed approach merges the above two methodological developments, i.e., the sNM3F algorithm and the task-decoding metric, into a single method that uses task information already at the stage of module computation. The rationale builds on the idea that an effective modular representation should not only allow reconstructing the original and noisy EMG data but should also guarantee discrimination of the performed motor tasks. Hence, instead of evaluating the decomposition in task space as a second verification step, we impose task discrimination explicitly during the extraction of modules. It is possible that inserting task categorization information directly into the first step of the module's computation could further improve either the compactness of the set of modules that carry task information or the overall task discriminability achieved in a decomposition, as proved by other machine learning studies for example in image or speech processing (Zafeiriou et al., 2006; Kotsia et al., 2007; Lee et al., 2012). Figure 1 provides a schematic illustration of how the method operates. For illustration, we present the activation of nine muscles during performance of three motor tasks (task1, task2, task3). Muscle activations are approximated by a lower-dimensional module space defined by spatial and temporal modules. Typical module extraction algorithms aim to extract the optimal modules that minimize the error obtained when reconstructing the single-sample muscle activations in the module space. The proposed method adds to this objective the task categorization objective to implement the premise that the single-sample module activations (colored dots representing 10 samples for each task in Figure 1 middle) should allow reliable discrimination of the performed tasks. Tasks are more discriminable if module activations are similar across samples for the same task and dissimilar between tasks. To achieve this formally, our method aims to minimize the within-task dispersion of module activations (illustrated by the green, red, and yellow ellipses) and maximize the dispersion of the between-task activations (illustrated by the dashed-line black ellipse). Hence, the method optimizes simultaneously the data approximation and the task discrimination objectives to identify invariant spatial and temporal modules as well as coefficients that combine them in single samples.

To implement the method, we develop a novel module identification algorithm, termed discriminative sample-based non-negative matrix tri-factorization (DsNM3F) that optimizes the extracted modules with two complementary yet simultaneous objectives: (a) to reconstruct the EMG data and (b) to discriminate the performed tasks. We propose three different implementations of DsNM3F, whose efficiency may depend on the data set under investigation. We illustrate the usefulness of the new algorithm in a simple simulation. We then apply it to EMG data with available task information and show its effectiveness in task categorization and data reconstruction.

## 2. Materials and methods

### 2.1. Experimental data set

The experimental data set that we will use throughout this study is composed of the EMG activity recorded from nine upper limb muscles during execution of arm pointing movements in the horizontal plane (see Delis et al., 2014 for full details on the experimental protocol). Six healthy right-handed subjects participated voluntarily in the experiment ad gave informed consent which was approved by the local ethical committee ASL-3 (“Azienda Sanitaria Locale”, local health unit), Genoa. The experiment conformed to the declaration of Helsinki. In short, each subject sat in front of a table and was instructed to perform center-out and out-center one-shot point-to-point movements between a central location (P0) and four peripheral locations (P1–P2–P3–P4) evenly spaced along a circle of radius 40 cm with either normal or fast speed. In total, the experimental protocol specified four targets × two directions, that is, eight distinct movement directions. Two speeds were tested, so that the 16 distinct motor tasks were denoted by *n*_1_,…, *n*_8_ and *f*_1_,…, *f*_8_ for normal and fast speeds, respectively. Each motor task was composed of 40 trials. Thus, we had a number of muscles *M* = 9 and a number of samples *S* = 16 × 40 = 640, for each participant. Here, we illustrate our method on the dataset recorded from one representative subject and then test it on all other subjects to assess its robustness.

Body kinematics was recorded by means of a Vicon (Oxford, UK) motion capture system. Six passive markers were placed on the finger tip, wrist (over the styloid process of the ulna), elbow (over the lateral epicondyle), right shoulder (on the lateral epicondyle of the humerus), back of the neck, and left shoulder. The kinematics data were low-pass filtered (Butterworth filter, cut-off frequency of 20 Hz) and numerically differentiated to compute tangential velocity and acceleration of the fingertip. Movement onset and movement end were identified as the times in which the fingertip velocity profile superseded 5% of its maximum. The mean movement duration varied across subjects from 370 to 560 ms. Electromyographic activity was recorded by means of an Aurion (Milan, Italy) ZeroWire wireless surface electromyographic system. The EMG signals were recorded from the following muscles: 1. finger extensors of the posterior forearm (extensor digitorum) (Fe), 2. brachioradialis (Br), 3. biceps brachii (Bi), 4. triceps medial (Tm), 5. triceps lateral (Tl), 6. anterior deltoid (Ad), 7. posterior deltoid (Pd), 8. pectoralis (Pe), 9. latissimus dorsi (Ld). EMG signals were digitized, amplified (20-Hz high-pass and 450-Hz low-pass filters), and sampled at 1000 Hz (synchronized with kinematic sampling). Subsequently, in order to extract the signal envelopes, the EMGs for each sample were digitally full-wave rectified, low-pass filtered (Butterworth filter, cut-off frequency of 3 Hz, zero-phase distortion; Ivanenko et al., 2004) and their duration was normalized to 50 time steps. The data were then normalized in amplitude on a muscle-per-muscle basis by dividing each single-sample muscle signal by its maximal value attained throughout the experiment. Thus, we finally formed an EMG matrix of dimensions (nine muscles×50 time steps) × 640 samples consisting of all the movement-related EMG activity (rectified and filtered) of the nine muscles for all recorded samples. This matrix will serve as a test input for the decomposition algorithms presented below.

### 2.2. Space-by-time decomposition model of muscle activity

To represent muscle activity as a structured modular decomposition, we used the model of concurrent spatial and temporal modularity in muscle activity presented in Delis et al. (2014). This space-by-time model decomposes muscle patterns into linear combinations of both spatial and temporal modules, and unifies the most usual types of models. The decomposition of a single-sample muscle pattern **m**^*s*^(*t*) ∈ ℝ^*T*_+_ × *M*^ can be written explicitly as follows (*T* and *M* being the number of time frames and muscles, respectively):

(1) $$m^{s}(t)=\sum_{i=1}^{P}\sum_{j=1}^{N}w_{i}(t)a_{ij}^{s}w_{j}+\text{residual},$$

where *w*_*i*_(*t*) ∈ ℝ^*T* × 1^_+_ and **w**_*j*_ ∈ ℝ^1 × *M*^_+_ are the temporal and spatial modules respectively, and *a*^*s*^_*ij*_ ∈ ℝ_+_ is a scalar activation coefficient. The free parameters *P* and *N* correspond to the number of temporal and spatial modules, respectively. For convenience, we define the matrix $A^{s}={\left(a_{ij}^{s}\right)}_{\begin{array}{c} 1\leq i\leq P\\ 1\leq j\leq N \end{array}}$.

More formally in matrix notation, the decomposition factors each single-sample non-negative muscle pattern as follows:

(2) $$M^{s}\approx \tilde{W}A^{s}W\;\forall s\in [1,S]$$

where $\tilde{W}$ ∈ ℝ^*T*_+_ × *P*^ is a matrix whose columns are the temporal modules, *W* ∈ ℝ^*N* × *M*^_+_ is a matrix whose rows are the spatial modules. Note that these matrices $\tilde{W}$ and *W* are independent of any particular sample/trial, and constitute the invariant modules necessary to synthetically describe the muscle activity in a variety of motor tasks (via the specification of activation coefficients *a*^*s*^_*ij*_).

### 2.3. Two-step module characterization

To identify the most compact and task-discriminative space-by-time decomposition, we took a two-step approach that we developed before Delis et al. (2013a,b). The first step consists in extracting spatial and temporal modules that approximate the original muscle activities and the second in selecting the set of modules that ensures the highest task discrimination. We detail these two steps below as they are relevant to the new method derived subsequently.

#### 2.3.1. NMF-based module extraction

To uncover the spatial and temporal modules, we used an NMF-based algorithm, called sNM3F (Delis et al., 2014). The sNM3F algorithm takes as input the preprocessed EMG matrix and the desired number of modules (*N* and *P*) and gives as output the matrices $\tilde{W}$, *A*^*s*^, *W* representing the spatial modules, sample-dependent activation coefficients, and temporal modules, respectively. The algorithm aims to reconstruct the recorded EMG data by minimizing the following NMF-based error, while imposing non-negativity constraints on $\tilde{W}$, *A*^*s*^, *W*:

(3) $$E_{\text{NMF}}^{2}=\sum_{s=1}^{S}||M^{s}-\tilde{W}A^{s}W|{|}_{\text{fro}}^{2}.$$

where ||.||_fro_ is the Frobenius norm.

The metric typically used to assess how accurately the modular decomposition reconstructs the original data is the Variance Accounted For (VAF) (d'Avella et al., 2006; Delis et al., 2013a):

(4) $$\text{VAF}=1-E_{\text{NMF}}^{2}/\sum_{s=1}^{S}||M^{s}-\bar{M}|{|}_{\text{fro}}^{2},$$

where *M* is the average muscle pattern computed across all the samples of the experiment.

#### 2.3.2. LDA-based module evaluation

We tested *a posteriori* whether the resultant decomposition allowed discrimination between the motor tasks performed in single-trial basis. Precisely, we quantified the task discrimination power of the identified modular decomposition using a previously developed task decoding procedure (Delis et al., 2013a). To predict the motor task executed in each sample, we used a linear discriminant algorithm (LDA) in conjunction with a leave-one-out cross-validation. As decoding parameters, we used the activation coefficients *a*^*s*^_*ij*_ recruiting the spatial and temporal modules in each sample (i.e., *N* × *P* parameters). We measured decoding performance as the percentage of correct predictions (DEC).

To select the dimensionality of the decomposition, we relied upon the decoding method introduced in Delis et al. (2013a, 2014). The core idea of the method is that decoding performance should significantly increase only if inclusion of an additional module describes reliably some task-related EMG variations not described by other already included modules. Hence, this procedure ensured the detection of modules that explain only the “task-relevant” variability and the exclusion of other sources of noise that produce “task-irrelevant” variability. This two-step process (NMF-based plus LDA-based) has been shown to effectively identify spatial and temporal modules that explain task differences in muscle activations (Delis et al., 2014). For the dataset under investigation, *P* = 3 temporal modules and *N* = 4 spatial modules were shown to be the optimal choice (see Results, Spatial and Temporal Modules).

### 2.4. Incorporating task discrimination in module extraction

The above approach has the potential drawback of separating the data approximation step from the task discrimination step in the module identification process. In fact, the modules are learned using an approximation criterion, whereas discrimination is employed for selecting the ones that convey the largest information about the tasks being performed and/or for verifying the task-discrimination power of the resultant decomposition. However, it is possible that making use of task discrimination during module extraction could lead to more discriminative decompositions while still giving a good approximation of the data. To test this, we developed a new method for learning spatial and temporal modules that merges the two above steps by imposing both approximation (NMF) and discrimination (LDA) objectives on the extracted modules, as proposed by Zafeiriou et al. (2006) or Kotsia et al. (2007) for instance.

First, in order to integrate task information into the decomposition, it is necessary to include in the optimization cost function a term related to task discriminability. This term will depend on the parameters that carry information about task differences, i.e., the single-sample activation coefficients (*A*^*s*^)_1≤*s* ≤ *S*_ of the decomposition. Here, we used a cost inspired by LDA and Fisher's criterion (Fisher, 1936).

We assume that the *S* samples can be partitioned in *K* classes (or tasks), $G=\cup_{k=1}^{K}$ *G*_*k*_ = ∪^*K*^_*k* = 1_ *G*_*k*_, where *n*_*k*_ denotes the number of samples belonging to the class *G*_*k*_, and $\sum_{k=1}^{K}n_{k}=S$. We defined the within-class and between-class scatter matrices as follows:

(5) $$\begin{array}{l} S_{w}=\sum_{k=1}^{K}\sum_{s\in G_{k}}\text{vec}(A^{s}-{\bar{A}}_{k})\text{vec}{\left(A^{s}-{\bar{A}}_{k}\right)}^{⊤}\\ S_{b}=\overset{K}{\underset{k=1}{\sum }}\text{vec}({\bar{A}}_{k}-\bar{A})\text{vec}{\left({\bar{A}}_{k}-\bar{A}\right)}^{⊤} \end{array}$$

where *Ā* and *Ā*_*k*_ denote the mean activation coefficient matrices across all the samples and across samples belonging to class *G*_*k*_, respectively, and vec denotes the vectorization. We denote by *n*_*l*_ the number of samples belonging to group *G*_*l*_. The matrix *S*_*w*_ defines the diffusion of sample vector coefficients around their class mean vec(*Ā*_*k*_). To increase discriminability across classes, the dispersion of samples that belong to the same class around their corresponding mean should be as small as possible. A convenient metric for the dispersion of the samples is the trace of *S*_*w*_, which has to be minimized. The matrix *S*_*b*_ denotes the between-class scatter matrix and defines the diffusion of the mean vectors of all classes around the global mean vec(*Ā*). As each class should be as far as possible from the other classes, the trace of *S*_*b*_ should be as large as possible. We linearly combined these two discrimination metrics and added them to the NMF-based approximation cost to build our total cost function that needs to be minimized during module extraction:

(6) $$E^{2}=E_{\text{NMF}}^{2}+\gamma \text{tr}(S_{w})-\delta \text{tr}(S_{b}).$$

where γ and δ are free tuning parameters and the negative sign of the last term indicates that *S*_*b*_ is maximized, and tr(.) denotes the trace operator. We will refer to the discrimination cost as LDA-related and define *E*^2^_LDA_ = γtr(*S*_*w*_) − δtr(*S*_*b*_).

### 2.5. Discriminative non-negative matrix tri-factorization for extraction of muscle activation modules

To solve the problem stated above, i.e., learn a modular decomposition that also attempts to maximize task discrimination, we developed and implemented three optimization algorithms. The first algorithm was based on the NMF multiplicative update rules (Lee and Seung, 1999), the second relied on constrained alternated least-squares (Berry et al., 2007) and the third method just formulated the problem as large-scale non-linear optimization (Liu and Nocedal, 1989; Byrd et al., 1999). We detail each one in turn.

#### 2.5.1. Multiplicative algorithm (MULT)

The update rules of the temporal and spatial modules were the same as for the sNM3F algorithm, as the LDA-related cost does not act on the modules:

(7) $$Q^{\prime }≜\tilde{W}A^{\prime }\text{and}\;W_{i,j}\leftarrow W_{i,j}\frac{{\left(Q^{⊤}ℳ\right)}_{i,j}}{{\left(Q^{⊤}QW\right)}_{i,j}}\forall i\in [1,N],j\in [1,M]$$

(8) $$ℛ≜AW\;\text{and}\;{\tilde{W}}_{i,j}\leftarrow {\tilde{W}}_{i,j}\frac{{\left({ℳ}^{\prime }ℛ\right)}_{i,j}}{{\left(\tilde{W}{ℛ}^{\prime }ℛ\right)}_{i,j}}\forall i\in [1,T],j\in [1,P]$$

where a calligraphic symbol ℳ denotes the vertical concatenation of sample elements *M*^*s*^ (a prime superscript denotes the horizontal concatenation, or “block transpose” operation).

The update rule for the activation coefficients had to be adapted to incorporate the task discrimination objectives. Calculating the gradient of the error for a given *A*^*s*^, it can be shown (see the Appendix in Supplementary Materials for details) that the new update rule for the activation coefficient is, supposing that the sample *s* belongs to the group *G*_*l*_ (with *n*_*l*_ elements):

(9) $$\begin{array}{l} A_{i,j}^{s}\leftarrow A_{i,j}^{s}\frac{{\left({\tilde{W}}^{⊤}M^{s}W^{⊤}+\frac{\delta }{n_{l}}{\bar{A}}_{l}+\gamma {\bar{A}}_{l}+\delta \frac{K}{S}\bar{A}\right)}_{i,j}}{{\left({\tilde{W}}^{⊤}(\tilde{W}A^{s}W)W^{⊤}+\frac{\delta }{n_{l}}\bar{A}+\gamma A^{s_{l}}+\frac{\delta }{S}\overset{K}{\underset{k=1}{\sum }}{\bar{A}}_{k}\right)}_{i,j}}\\ \;\forall i\in [1,P],j\in [1,N] \end{array}$$

At each iteration, the rows of $\tilde{W}$ and the columns of *W* are also normalized to sum to one. This is a necessary constraint of the problem formulation to avoid diverging behavior of the activation coefficients. While this normalization is not an issue for the standard sNM3F algorithm (Delis et al., 2014), here adding this constraint does not ensure anymore that the error is non-increasing at each iteration. Even though we found a good convergence of this algorithm in practice, we also considered alternative implementations to check the consistency of the results given by the algorithm.

#### 2.5.2. Alternating constrained least-squares (ALS)

We consider an alternative formulation to specifically preserve the normalization constraint during the update of $\tilde{W}$ and *W*. To this aim, we considered a constrained optimization problem.

(10) $$\underset{\tilde{W}}{\min}||ℳ-\tilde{W}ℛ|{|}_{\text{fro}}^{2}\text{s}.\text{t}.\sum_{i=1}^{T}{\tilde{W}}_{ij}=1\forall j\in [1,P]$$

After vectorization using Kronecker products, this can be rewritten as:

(11) $$\left(\begin{array}{cc} 2ℛ{ℛ}^{⊤}⊗I_{T} & I_{P}⊗1_{T}\\ I_{P}⊗1_{T}^{⊤} & 0 \end{array}\right)\left(\begin{array}{c} \text{vec}(\tilde{W})\\ \lambda \end{array}\right)=\left(\begin{array}{c} \text{vec}(2ℳ{ℛ}^{⊤})\\ 1_{P} \end{array}\right)$$

where **1**_*P*_ is a vector of *P* ones and ⊗ denotes the Kronecker product.

This is a standard system of linear equations for which a least-square solution can be efficiently obtained (e.g., *mldivide* Matlab function).

Similarly, to update *W*, one can solve the following least-square problem:

(12) $$\left(\begin{array}{cc} I_{N}⊗2Q^{\prime ⊤}Q^{\prime } & 1_{M}⊗I_{N}\\ 1_{M}^{⊤}⊗I_{N} & 0 \end{array}\right)\left(\begin{array}{c} \text{vec}(W)\\ \lambda \end{array}\right)=\left(\begin{array}{c} \text{vec}(2Q^{\prime ⊤}{ℳ}^{\prime })\\ 1_{N} \end{array}\right).$$

Since non-negativity is not ensured after such a process, it can be imposed *a posteriori* by setting all negative elements to zero.

Alternatively, a quadratic programming problem could be solved by imposing non-negativity constraints on the elements of $\tilde{W}$ and *W*, which ensures a decreasing error but may slow down the algorithm significantly.

(13) $$\begin{array}{ccc} \min_{\tilde{W}}||ℳ-\tilde{W}ℛ|{|}_{\text{fro}}^{2} & & \text{s}.\text{t}.\sum_{i=1}^{T}{\tilde{W}}_{ij}=1\forall j\in [1,P]\\ & & \text{and}\;{\tilde{W}}_{ij}\geq 0\forall i\in [1,T],j\in [1,P] \end{array}$$

#### 2.5.3. Non-linear programming (NLP)

To derive our third method, we formulated the problem as a single large-scale constrained optimization problem. We implemented the gradients of the cost function and of the non-linear constraints to perform the actual optimization using *fmincon* Matlab function with calculation of the Hessian by a limited-memory, large-scale quasi-Newton approximation (namely, the interior-point algorithm with *lbfgs* method).

The NLP problem was thus formulated as:

(14) $$\underset{{}_{z}}{\min}E^{2}(z)\text{s}.\text{t}.z_{i}\geq 0\forall i\in [1,TP^{2}N^{2}SM]\text{and}\phi (z)=0.$$

where $z^{T}≜(\text{vec}{\left(\tilde{W}\right)}^{⊤},\text{vec}{\left(A\right)}^{⊤},\text{vec}{\left(W\right)}^{⊤})$, i.e., the concatenation of the vectorizations of $\tilde{W}$, all the *A*^*s*^, and *W*. The function ϕ encoded the additional constraints to normalize the rows of $\tilde{W}$ and columns of *W* so that they summed to one (linear constraint). Comparisons of the three methods revealed that they were effective and yielded the same modular decompositions. The best choice of algorithm implementation (in terms of speed and robustness) may depend on the data set, but overall the MULT algorithm yielded good performance. Matlab code implementing DsNM3F including these three optimization algorithms is available at the last author webpage (http://hebergement.u-psud.fr/berret/software/DsNM3F.zip).

### 2.6. Comparison between decompositions

We defined *VDM* = *VAF*× *DEC* (0 ≤ *VDM* ≤ 1), a measure of the goodness of an EMG decomposition that incorporates the VAF (data approximation) and the decoding performance (task discrimination). Higher values of VDM correspond to more plausible decompositions that achieve the best trade-off between data approximation and task discrimination. Hence, we used this metric to select the best output of DsNM3F with respect to parameters γ and δ, and compare it to standard space-by-time decomposition identified by sNM3F.

To assess the similarity between modules identified by DsNM3F and sNM3F, we used as similarity index the correlation coefficient between pairs of modules (either spatial or temporal). To compare activation coefficients, we computed the average coefficients for each of the 16 tasks, which gave a 16-dimensional vector for each decomposition and computed the correlation coefficients for pairs of such vectors.

To assess the power of a modular decomposition in terms of task prediction, we used Support Vector Machine (SVM) classification (implemented in Matlab using the LIBSVM toolbox Chang and Lin, 2011). We applied linear SVM to the activation coefficients combined with a repeated random subsampling cross-validation procedure (Cortes and Vapnik, 1995; Theodoridis and Koutroumbas, 2008). More precisely, we split the dataset into training and test data 20 times making sure that both training and test data had always the same task proportions. More precisely, we tested the cases of 5, 10, and 30 training samples per task. For each one of the 20 splits, first we applied the module identification algorithms (sNM3F or DsNM3F) to the training data. Then, we kept only the extracted modules since they represented the basic modules on which the muscle patterns could be projected. Therefore, we projected all the data (both training and test) on them without taking into account task information (as it was anyway unavailable for the test data). To do this, we used the sNM3F with fixed spatial and temporal modules, i.e., updating only the activation coefficients. This procedure gave us new activation coefficients for both the training and test data sets. We used the activation coefficients of the training dataset to train a linear SVM classifier and the ones of the test dataset to compute the classification performance (percentage of correctly classified samples). We repeated this process 20 times and computed the average (±SD) classification score (denoted by CLS).

## 3. Results

To illustrate the newly developed DsNM3F algorithm and have a better understanding of its functionality, first we tested it on simulated data for which the modules and their activations are known by construction. Then, we applied to the complete EMG dataset recorded during performance of 16 arm pointing tasks (40 trials per task), which has been analyzed before using the space-by-time decomposition model (Delis et al., 2014). Both datasets allowed us to make direct comparisons between the outputs of DsNM3F and sNM3F.

### 3.1. Trade-off between task discrimination and data approximation

**Figure 2:**
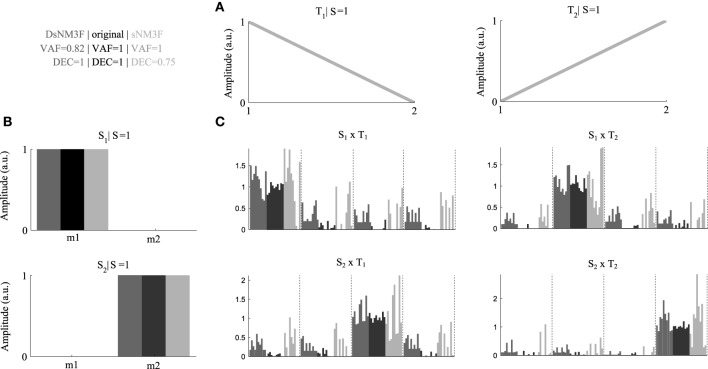
**Performance comparison DsNM3F and sNM3F on simulated data**. **(A)** Two temporal modules were used to generate the data and were recovered correctly by both algorithms. **(B)** Two spatial modules were used to generate the data and were recovered correctly by both algorithms. **(C)** We simulated coefficients combining the spatial and temporal modules to perform ten repetitions of four hypothetical tasks. Black bars correspond to the simulated coefficients over the ten trials for each task DsNM3F reconstructed the activations (dark gray bars) more accurately than sNM3F (light gray bars). Dashed vertical lines separate different tasks.

To exemplify the usefulness of the new algorithm, we generated an artificial EMG dataset consisting of two spatial modules and two temporal modules (Figures 2A,B) and simulated their activations during performance of four motor tasks, each repeated ten times with relatively small inter-trial variability (Figure 2C, black bars) that allows perfect task discrimination. For simplicity, we modeled the spatial modules to consist of two muscles and the temporal modules to consist of two time points. The EMG signals (constructed by combining spatial and temporal modules using the single-trial activations) were corrupted with large additive and multiplicative noise (40% of the signal) to model different sources of measurement noise in the EMG recordings. We applied the standard sNM3F and the new DsNM3F to the resulting EMG datasets and found that both algorithms reconstructed accurately the original spatial and temporal modules despite the presence of significant noise (Figure 2, dark gray for DsNM3F, light gray for sNM3F, and black for original modules). DsNM3F approximated the original activation coefficients more accurately than sNM3F indicating that the insertion of the task discrimination objective resulted in a better reconstruction of the task-dependent structure of the module activations. This result is also reflected in the VAF and DEC values of the two decompositions. In light of the data generation, an imperfect VAF is expected even if both the modules and the true activation coefficients are correctly recovered because of the addition of unstructured noise to the EMG signals. In contrast, this noise should not affect the task-dependence of the activation coefficients (i.e., perfect task decoding if they are well identified). Here, DsNM3F achieved perfect task categorization and lower VAF. However, sNM3F achieved maximal VAF at the expense of task discrimination power. The reason is that the only objective of sNM3F is data reconstruction so that it attempts to recover all the variability in the data (even the one that is due to measurement noise), which therefore degrades the reconstruction of the task dependence of the activations. This can be viewed as overfitting in the module activations space. The insertion of task discrimination objectives in DsNM3F is a way to address this issue by balancing data approximation with task discrimination. Hence, DsNM3F appears to be useful for identifying representations that achieve better VAF-DEC trade-offs and may thus be more plausible in task space.

### 3.2. Influence of parameters on VAF and task decoding

**Figure 3:**
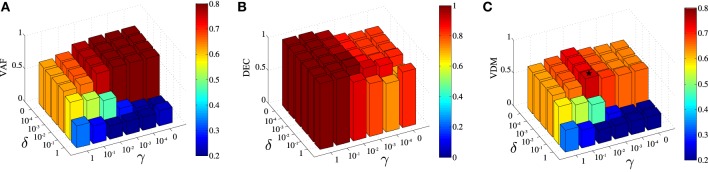
**Influence of the parametersγ and δ on the extracted space-by-time decomposition. (A)** Dependence of the Variance Accounted For (VAF) on γ and δ. VAF takes higher values for low values of the two parameters. **(B)** Dependence of task classification (DEC) on γ and δ. DEC takes higher values for high values of γ (mainly) and δ. **(C)** The VAF-Decoding metric (VDM) is defined as the product of the two metrics in **(A,B)**. The star symbol depicts the values of the pair of parameters γ and δ that maximize VDM, here (γ, δ) = (0.01, 0.01).

DsNM3F has two free parameters γ and δ that need to be carefully selected in order to achieve the optimal solution that balances task discrimination with data approximation. In our framework, we measure task discrimination as percent correct decoding performance (DEC) and data approximation as the VAF by the decomposition. As our goal is to achieve the highest possible task discrimination without a significant data approximation loss, we chose to maximize VDM, a metric that incorporates both objectives (see Materials and Methods for details). To determine the optimal pair of parameters, we developed an automated optimization procedure. First, we used a coarse grid in (δ,γ)-space to find an approximate value maximizing VDM. Second, we ran an optimization starting from the latter initial guess in order to tune more finely the parameters and try to get the best possible VDM value. Typically, this required running the DsNM3F with varying values of γ and δ. Note that throughout the paper, we always chose the best modular decomposition among a large number of restarts (~100) to limit the problem of local minima and reason on reliable factorizations. We used the output to compute VAF, DEC, and in turn VDM (see Materials and Methods). In Figure 3, we show the dependence of VAF, decoding and VDM on γ and δ for the initial coarse grid. The highest VAF is obtained for γ = δ = 0 as expected, which corresponds to removing the discrimination objective from the optimization (equivalent to standard sNM3F). The VAF is high for small values of γ and δ and, as expected, decreases with values of both parameters close to 1 (i.e., when more weight is put on discrimination). Conversely, decoding performance is high for large values of γ and δ and decreases as their values decrease. Interestingly, decoding shows a higher dependence on γ than δ indicating that decreasing the within-task variability plays a bigger role in decoding performance than increasing the between-task variability. The maximal VDM appears as a compromise between the two metrics and corresponds to γ = 0.01 and δ = 0.01 (illustrated with a star on the graph). These parameter values were actually very close to the global optimum (the best solution we found was for (γ, δ) = (0.01, 0.006) giving a VDM value very close to the one found using the coarse grid procedure), which finalizes our process for finely tuning the two free parameters. This approach was successful in determining the optimal values of γ and δ also for the data recorded from the remaining five subjects. Parameter values were found to be quite robust across datasets: (γ, δ) = (0.01,0.01), (0.0001,0.1), (0.01,0.01), (0.01,0.01), (0.0001,0.01) for the five other subjects, respectively.

### 3.3. Spatial and temporal modules

**Figure 4:**
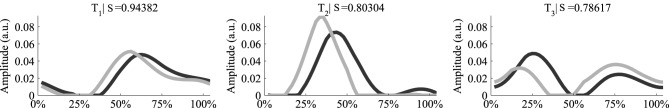
**Comparison of the three temporal modules extracted by sNM3F (black) and DsNM3F (gray)**. The temporal modules are waveforms over the time course of movement and are ordered by similarity (index *R*) here. Overall the temporal modules appear to be highly similar in the two cases.

**Figure 5:**
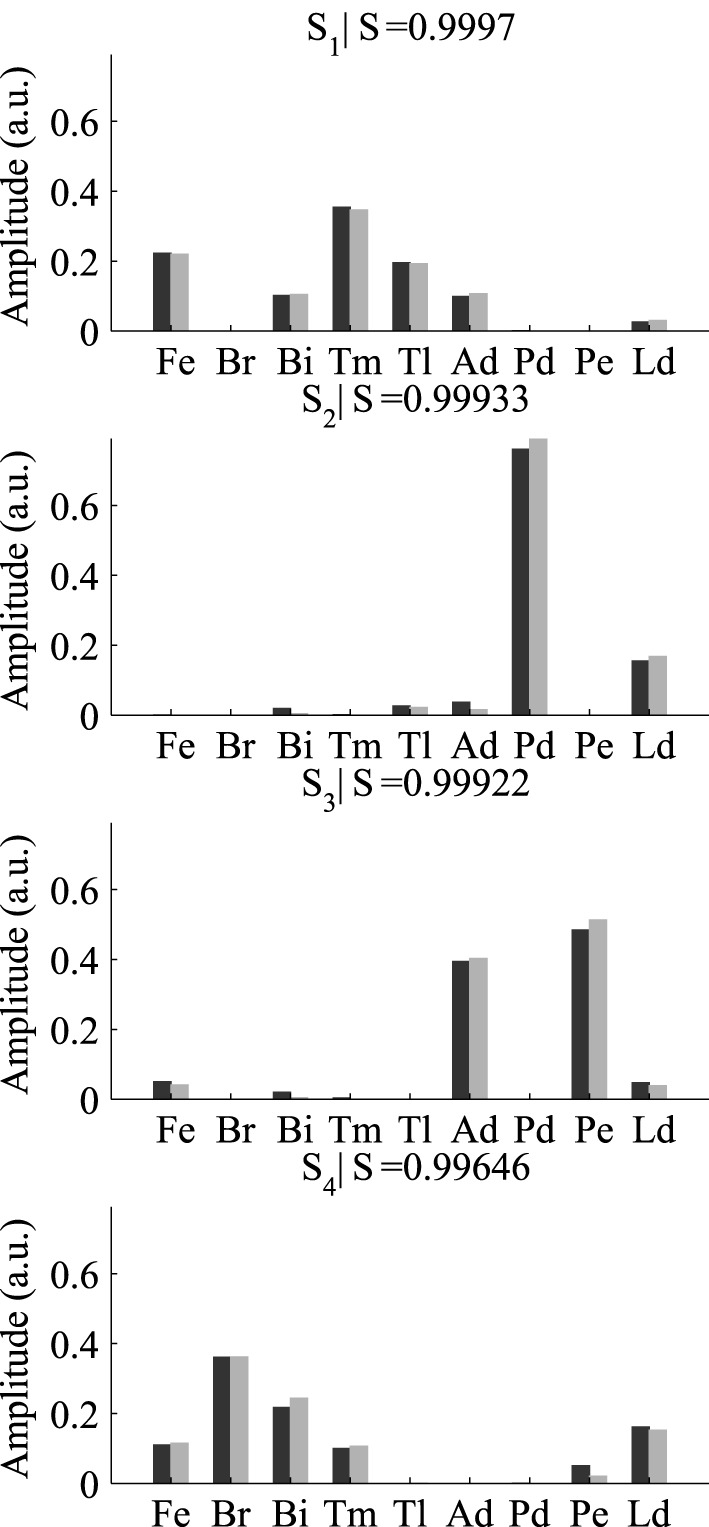
**Comparison of the four spatial modules extracted by sNM3F (black) and DsNM3F (gray)**. The spatial modules are weightings of muscle activations and are ordered by similarity (index *R*). Overall the spatial modules appeared to be almost identical in the two cases.

After selecting the best γ and δ, we used these optimal parameter values and ran the DsNM3F algorithm to decompose the EMG data into spatial and temporal modules. In a previous study (Delis et al., 2014), we showed that three temporal and four spatial modules constitute the lowest-dimensional decomposition that achieves the highest decoding performance. Thus, we used the same temporal and spatial dimensionality in this study and verified whether decoding could not be increased more. Figure 4 shows the temporal modules extracted by DsNM3F (gray) and the ones extracted by sNM3F (black). The variable *R* indicates the similarity between pairs of modules. The two decompositions yielded highly similar temporal modules consisting of successive bursts of muscle activity (*R* > 0.75). Both decompositions identified two modules with a single activation burst and a third one composed of two bursts. These temporal decompositions are reminiscent of the well-known triphasic pattern of muscle activity observed during the single-joint rotations but also during whole-body actions: a first temporal burst activates agonist muscles to initiate the movement, a second temporal burst activates antagonist muscles to decelerate the movement and a last burst activates again agonist muscles to stabilize over the endpoint (Berardelli et al., 1996; Chiovetto et al., 2010). The existence of a double-burst temporal module in our temporal decomposition (with an early and a late peak) likely signifies the activity of muscles that support the arm at the starting point (early activation) and co-contract to achieve precision at the endpoint (late activation). Figure 5 shows that the spatial modules extracted by the two algorithms were almost identical (*S* > 0.99). The spatial modules reveal four muscle groupings with distinct functional roles: elbow extensors (S1), shoulder flexors (S2), shoulder extensors (S3), and elbow flexors (S4).

When applying the two algorithms on the EMG data of the remaining five subjects, we found that the above results are robust across subjects. The two algorithms identified highly similar spatial modules (average similarity *R* = 0.97, 0.96, 0.99, 0.98 for the four modules, respectively) and also highly similar temporal modules (average similarity *R* = 0.95, 0.91, 0.97 for the three temporal modules, respectively).

### 3.4. Activation coefficients

**Figure 6:**
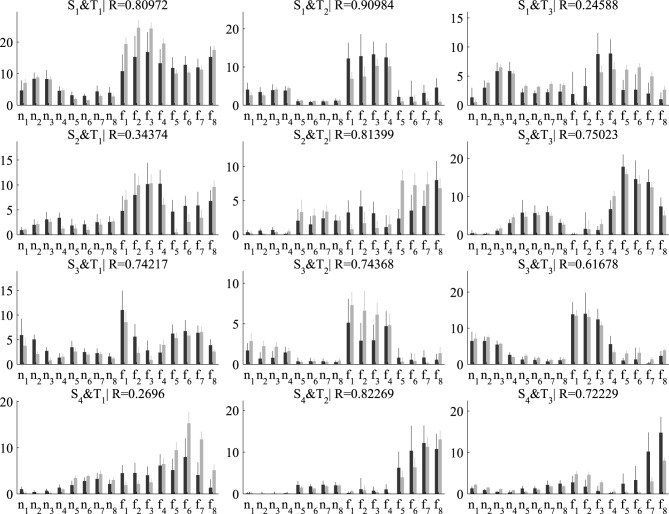
**Comparison of the activation coefficients combining the four spatial (in rows) and three temporal modules (in columns) extracted by sNM3F (black bars) and DsNM3F (gray bars)**. Each bar indicates the mean ± SD of all the samples for each task (*n* and *f* stand for normal and fast speeds, respectively; subscripts indicate the movement direction). The two algorithms show differences in the activation coefficients: (a) an overall decrease of the inter-sample variability for the DsNM3F (smaller gray error bars than black error bars for all coefficients and tasks) as well as (b) modifications of the average activation coefficients (e.g., small *R*-values for *S*_2_ × *T*_1_ and *S*_4_ × *T*_1_).

Next, we examined the coefficients that combine the spatial and temporal modules in single samples. For our decompositions consisting of three temporal and four spatial modules, the number of single-sample coefficients is 3×4 = 12. In Figure 6, we plot the average (± standard deviation) activation coefficients for each one of the 16 tasks performed in the experiment. Activation coefficients are higher for the eight fast speed tasks (denoted as *f*_1_,…, *f*_8_) compared to the eight normal speed tasks (*n*_1_,…, *n*_8_) for both algorithms, which indicates overall higher muscle activity for fast movements. Figure 6 also illustrates the task specificity of module recruitment. For example, the second temporal module (middle column in Figure 6) is combined mainly with the first and third spatial modules (elbow extensors and shoulder extensors, respectively) to perform tasks *n*_1_, *n*_2_, *n*_3_, *n*_4_ and *f*_1_, *f*_2_, *f*_3_, *f*_4_, whereas it is combined with the second and fourth spatial modules (shoulder flexors and elbow flexors, respectively) to perform tasks *n*_5_, *n*_6_, *n*_7_, *n*_8_ and *f*_5_, *f*_6_, *f*_7_, *f*_8_. Regarding differences between the two algorithms, the variability of the DsNM3F coefficients is lower compared to the sNM3F coefficients for all tasks (see the error bars in Figure 6). This decrease of the within-task variability of the activation coefficients results from the within-task variability penalization introduced into the objective function of the DsNM3F algorithm. Similarly, as illustrated by the similarity indices, the average activation coefficients extracted by the two algorithms exhibit considerable differences for some pairs of modules (see the low *R*-values for *S*_1_ and *T*_3_ and *S*_4_ and *T*_1_ for instance). This is likely a result of the between-task variability constraints that attempt to make average activations for each task as discriminable as possible from all other tasks. In sum, the effect of both within-task and between-task objectives was qualitatively observable in the results even though this did not imply any obvious modification of the underlying modules. This module invariance was mainly a result of imposing the data approximation objective. In fact, we noticed that the modules could change as well for larger values of γ and δ but at the price of considerably lowering VAF and therefore VDM. Hereafter, we assess these main observations quantitatively.

### 3.5. Effects of inserting a discrimination objective into sNM3F

To gain more insights into task separability and verify the effectiveness of the DsNM3F, we computed *tr*(*S*_*w*_) and *tr*(*S*_*b*_) that quantify the within-task and the between-class dispersion, respectively. For sNM3F, tr(*S*_*w*_) = 3.0e4 and tr(*S*_*b*_) = 2.4e3, whereas for DsNM3F tr(*S*_*w*_) = 7.1e3 and tr(*S*_*b*_) = 2.9e3. Thus, the overall within-class variance was much smaller for DsNM3F, which reflects onto a decrease of the inter-sample variance of the 16 activation coefficients [3.18±1.78 for sNM3F vs. 1.26±0.63 for DsNM3F (mean±SEM)]. Also, the between-task variability was higher for DsNM3F compared to sNM3F indicating that DsNM3F achieved higher task-to-task separation on average.

To summarize the above findings in one measure, we computed the ratio of the above quantities *J* = *tr*(*S*_*w*_)/*tr*(*S*_*b*_), which is an objective function to be minimized for maximal task discrimination. This was 2.49 for the DsNM3F and 12.3 for sNM3F, thus showing the task discrimination gain obtained when applying the DsNM3F algorithm to the complete dataset. This discrimination gain was also observed in the remaining five datasets we tested [24±2.6 for DsNM3F vs. 36±7.8 for sNM3F (mean±SEM)] and was achieved because activation coefficients were better clustered together for each task but also because the task centroids were moved toward more optimal locations in the space of activation coefficients. Applying LDA to the activation coefficients of the two algorithms gave 0.8 correct classification for sNM3F vs. 1 (i.e., perfect classification) for DsNM3F for the example subject and 0.675±0.06 vs. 0.933±0.03 on average (±SEM) across subjects. Hence, by modifying the single-sample activation coefficients, DsNM3F achieved significantly higher task separability. Importantly, this was possible with only a small decrease in data approximation [VAF was 0.797 for sNM3F vs. 0.770 for DsNM3F for the example subject and 0.766±0.02 vs. 0.712±0.03 on average (±SEM) across subjects]. In sum, VDM values [0.638 for sNM3F vs. 0.770 for DsNM3F for the example subject and 0.518±0.043 vs. 0.656±0.029 on average (±SEM) across subjects] indicate that DsNM3F achieves overall a better trade-off between task discrimination and data approximation. If we combine these results with the finding that DsNM3F alters minimally the extracted spatial and temporal modules, we conclude that DsNM3F increases task discrimination by optimizing mainly the activation coefficients and disregarding better task-irrelevant measurement noise in that space.

### 3.6. Predictive power of the decompositions

So far, we have shown that the new DsNM3F algorithm allows almost perfect task discriminability when applied to data for which task information is already available. A subsequent question is to examine whether the algorithm can be used for prediction, i.e., to test how accurately it decodes the task for independent samples corresponding to unknown tasks. This problem requires the computation of a dedicated projection mapping from the EMG data onto the task-dependent activation coefficients computed by DsNM3F. It is likely that this mapping cannot be made by means of a simple projection onto the extracted modules because, as we showed above, they do not really carry the extra task information introduced by the task discrimination objectives. Nevertheless, as there are slight differences in the extracted temporal modules, we can still test how DsNM3F compares with sNM3F when no task information is available.

**Figure 7:**
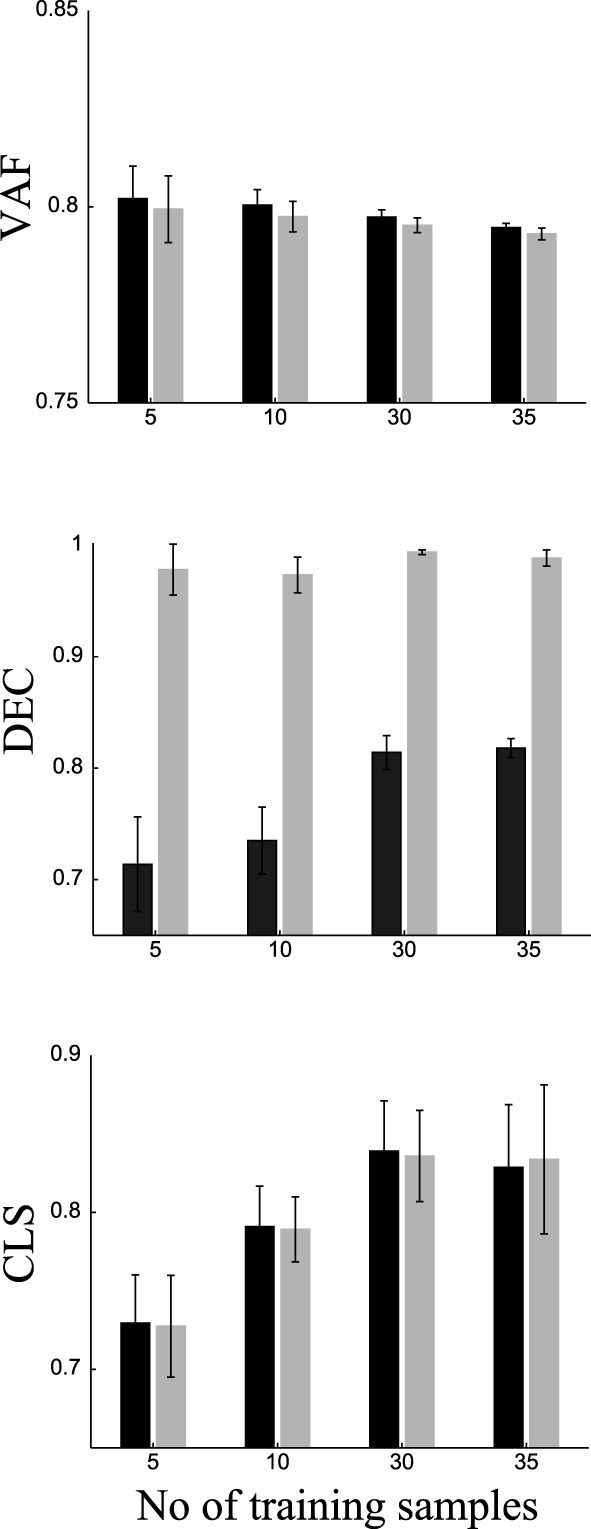
**Comparison of VAF, DEC (classification on trained data) and CLS (classification on independent test data) for sNM3F (black bars) and DsNM3F (gray bars) varying the number of training samples for each task**. Each bar indicates the mean ± SD over 20 repetitions of random subsampling cross-validation.

To do this, we randomly split our data into two sets of samples. We exploited task information only in the first set (training samples) and used the second set (test samples) to predict the task of each sample (see Materials and Methods for a detailed explanation of the procedure). We repeated this procedure 20 times using different random splits and averaged VAF, DEC, and classification scores (CLS) over repetitions. To assess the importance of training on these results, we varied the number of training samples from each task (namely 5, 10, 30, and 35). The results are shown in Figure 7. VAF for DsNM3F is slightly but not significantly (*p* > 0.1) lower than for sNM3F for all training set sizes. For both algorithms, slightly lower VAF and more robust estimates of it (as indicated by the smaller error bars) are obtained when the number of training samples increases. Regarding DEC scores, i.e., classification performance on trained samples, DsNM3F achieves close to perfect classification for any training set size, whereas for sNM3F DEC is significantly lower and increases with the number of training samples (it saturates for 30 training samples at 0.815). Again, the variability of estimates decreases with the number of training samples. In sum, these results confirm that the insertion of discrimination objectives increases significantly task classification on trained data, while it does not decrease significantly VAF scores.

DsNM3F has equal performance with sNM3F in task classification when using independent new data. This finding can be explained in view of the fact that the two algorithms extract essentially almost the same modules and the projection on the modules does not exploit the learned distribution of activation coefficients. Since the modules constitute the sample-independent basis on which any newly acquired data can be projected and the two sets of basis functions are almost identical, the activation coefficients obtained by projecting the new data onto the modules (using a data approximation criterion alone) will be very similar too. Thus, there will be no difference in the resulting decoding performance. For both algorithms, decoding performance increases with the number of training samples and reaches its maximum for 30 samples from each task (no increase beyond that point). Interestingly, to perform reliable task prediction, a minimum of 30 training samples from each task is required. Taken together, these results indicate that the modules extracted by sNM3F or DsNM3F constitute the best possible basis with respect to task discrimination and data approximation.

## 4. Discussion

In this paper, we proposed a principled method for identifying data-approximating and task-discriminating modules of muscle activity. We introduced a new algorithm, named DsNM3F, which is an extension of a previously developed method (sNM3F algorithm) that extracts space-by-time representations of EMG data, i.e., decomposes EMG data into concurrent spatial and temporal modules and combines them in single samples (Delis et al., 2014). The novelty of our approach resides in the insertion of task discrimination objectives within the space-by-time module identification process.

### 4.1. Foundations of the new approach

The functional role of modularity in muscle activity consists in the accomplishment of a variety of motor tasks in single samples using a small fixed set of modules. Hence, for a modular representation to be functional and plausible, we assert that the extracted modules should not only reconstruct the recorded EMG activity as accurately as possible, but also categorize it with respect to the performed motor tasks. In the literature thus far the latter objective has typically been considered only after module identification. A number of studies have related the extracted module activations to task parameters (see e.g., Brochier et al., 2004; Torres-Oviedo and Ting, 2010; Chvatal et al., 2011). Other studies proposed computing VAF within each task separately to assess whether all tasks are accounted for by the decomposition (Torres-Oviedo and Ting, 2010; Chvatal et al., 2011; Roh et al., 2011) but did not explicitly assess task separability. Yet another approach proposed an algorithm for extracting task-specific modules (Cheung et al., 2009). This approach is indeed useful when the examined dataset contains tasks whose execution relies on synergies that are not shared with other tasks, however, it tends to increase the dimensionality of the extracted set of modules. Finally, in our previous work that motivated this study, we employed a single-sample task decoding metric for selecting the dimensionality of the set of modules and comparing alternative decomposition models (Delis et al., 2013a,b, 2014).

On a similar vein, novel approaches have been proposed to critically test the functional role of modularity in task space. Recent studies examine the use of modularity for task control and its ability to explain motor behavior (Kuppuswamy and Harris, 2014). In particular, modular decompositions have been shown to introduce errors in task accomplishment and depend on the dimensionality of muscle space as well as task space (de Rugy et al., 2013; Steele et al., 2013). Also, another study found that the current formulations of modular control were not fully adequate to explain muscle coordination in multidirectional locomotion tasks (Zelik et al., 2014). Our current formulation, as well as most formulations of modular control, implicitly involves an open-loop control scheme. While motor planning may rely on such a compact motor representation to generate genuine yet approximate muscle patterns, motor execution is known to rely on a closed-loop control scheme. Therefore, feedback may modify the on-line recruitment of modules or even the activity of single muscles (Valero-Cuevas et al., 2009). Hence, it is likely that the role of feedback should be modeled when considering task execution from modular decompositions. For instance, in Neptune et al. (2009), the timing of empirical modules had to be slightly modified to allow normal walking of a detailed musculoskeletal model of the lower limbs. In the present study, we only focused on the motor planning stage, where task discrimination is especially relevant but additional work is needed to understand the extent to which muscle pattern adjustments are critical for accurate task execution.

In this study for the first time we devised an algorithm, DsNM3F, that offers the possibility to compute modules in task space by combining task discrimination with data approximation objectives during the module identification process. We also introduced a new metric, VDM, whose maximization corresponds to an optimal trade-off between these two objectives and showed that we can reach such a trade-off by appropriately tuning the free parameters γ and δ of DsNM3F.

### 4.2. Insights about the space-by-time decomposition

We applied DsNM3F to simulated data with known underlying structure and to an EMG dataset collected during an arm pointing experiment that we studied before. For both datasets, we were able to compare with the standard sNM3F algorithm and gain valuable insights concerning the functionality and usability of the new method. Indeed, the DsNM3F algorithm validated previous results regarding the spatial and temporal structure of muscle activity. Interestingly, adding the task discrimination objective had little or no effect on the extracted spatial and temporal modules on condition that data approximation had to be preserved as much as possible. A possible explanation for this similarity was that even for DsNM3F the data approximation objective prevailed over the task discrimination objective, so it did not allow the emergence of alternative modules. In fact, we tested this by relaxing the data approximation constraint and found that it led to the identification of relatively different modules (data not shown). However, these new modules accounted for significantly lower variance and had lower task discrimination performance. Hence, we suggest as a more plausible explanation that the standard modules (extracted by either sNM3F or DsNM3F) constitute the lowest-dimensional representation that achieves the best VAF-DEC trade-off for this dataset. In this respect, our results support effectiveness of the space-by-time decomposition of muscle activity in finding robustly and consistently modules that carry task-relevant information, independently of the details of module computation.

Regarding module activations, our simulations suggested that DsNM3F retrieves the task dependence of the activation coefficients more accurately than sNM3F, which results in better task categorization. Indeed, when applied to the real data, the activations extracted by DsNM3F gave almost perfect task categorization. These findings suggest that DsNM3F should be the algorithm of choice when the aim is to distinguish between motor tasks and characterize how module activations are modulated by differences in task parameters (e.g., speed, amplitude, or direction tuning of module recruitment (e.g., d'Avella et al., 2008).

We also examined whether our results could generalize to new data with no task information and found that DsNM3F had equal performance with sNM3F. It is possible that better task prediction could be achieved by DsNM3F if the knowledge about the distribution of activation coefficients could be used for prediction. Indeed, we observed that the high decoding scores of DsNM3F mainly resulted from a rearrangement and clustering of the activation coefficients. To exploit this distribution, we need to learn a (possibly non-linear) projective mapping between the EMG data and the extracted activation coefficients. The standard NMF projection we used here is simply based on data approximation criteria and depends only on the shape of the spatial and temporal modules, thus it ignores the distribution of activation coefficients. In future work, it would be interesting to develop methods that perform this projection efficiently in order to enhance the generalization power of our approach. For example, neural networks or Gaussian processes could be used to learn how to map EMG data (or parts of such data) to task-discriminant activation coefficients. Knowing this mapping, new EMG data could be mapped onto the identified activation coefficients, which, in turn, would give both the task being performed and a predictive reconstruction of the full EMG pattern that should be used to execute that task. This type of approach may be useful in body-machine interfaces and myoelectric control devices for inferring user's intention when performing different goal-directed movements. We believe that learning such a mapping constitutes an additional interesting research question, which may be application-dependent and therefore will be treated in future work.

We suggested before that the space-by-time decomposition of muscle activations is not only task-relevant but also low-dimensional. We explicitly demonstrate this point using the dimensions of our experimental dataset as an example. To specify the activity of *M* muscles (*M* = 9 in our data) over *T* time frames (this value could be considered smaller as a result of filtering or larger to account for arbitrary waveforms in continuous time) for the execution of *S* movements (*S* = 640 = 16 tasks ×40 repetitions), the number of parameters to be determined for the whole dataset is *MTS* = 288,000. When applying the space-by-time decomposition to this dataset, the model parameters comprise (a) the activation coefficients combining the *N* spatial with *P* temporal modules to perform each movement *S*, i.e., *NPS* = 3×4 = 7680 parameters and (b) the spatial and temporal modules that need to be stored and reused across movements, i.e., *NM* + *TP* = 27 + 150 = 177 parameters, which makes 7857 parameters in total. Hence, for this example the space-by-time model of modularity reduces considerably the number of dimensions from 288,000 to 7857 (see also Delis et al., 2014).

At last, to investigate the above questions further, we also plan to apply the space-by-time decomposition using both proposed algorithms to other EMG datasets recorded during more complex motor tasks, in which task decoding will perhaps be more challenging, thus more differences between DsNM3F and sNM3F may be revealed.

### 4.3. Possible extensions of the algorithm and future work

A limitation of our approach is that the proposed algorithm works only with discrete motor tasks. In fact, our experimental protocol specified movement endpoints and speeds that define 16 distinct motor tasks. Future work on DsNM3F may consider extending the method to an unfixed number of tasks and also treating continuous task parameters, such as reach endpoint coordinates, movement trajectories, joint angle displacements etc. Other extensions of the space-by-time NMF decomposition may involve adding other constraints such as enhancing sparsity of the extracted spatial and temporal modules (Hoyer, 2004).

Finally, we indicate two possible avenues for future investigations. First, the use of generative models, such as non-negative restricted Boltzmann machines Downs et al., 2000), for extracting modules of muscle activity could be relevant for learning task-discriminative modular structures as this method was shown to have discriminative properties (Nguyen et al., 2013). Second, modular decompositions of muscle activity can be useful to assist motor rehabilitation or skill acquisition training (Safavynia et al., 2011; d'Avella et al., 2013). Development of methods that identify modules online, i.e., during the execution of motor tasks, could be exploited to obtain faster recovery of functional module recruitment.

## Grants

We acknowledge the financial support of the SI-CODE project of the Future and Emerging Technologies (FET) programme within the Seventh Framework Programme for Research of The European Commission, under FET-Open grant number: FP7–284553. This work was also supported by the Conseil Régional de Bourgogne, projet Pari “Correlats Neuronaux.”

### Conflict of interest statement

The authors declare that the research was conducted in the absence of any commercial or financial relationships that could be construed as a potential conflict of interest.
