# Supplementary material for: Task-discriminative space-by-time factorization of muscle activity
Source: Front Hum Neurosci. 2015 Jul 10;9:399. doi: 10.3389/fnhum.2015.00399 (PMC4498381; doi:10.3389/fnhum.2015.00399)
Supplement: Supplementary file 1 [file Appendix.PDF]

# Appendix

**Multiplicative rule** Here we show how to obtain the multiplicative update rule for the activation coefficients. We first note that $\mathrm{tr}(S_w) = \sum_{k=1}^{K} \sum_{s \in G_k} tr\big((A^s - \bar{A}_k)^\top (A^s - \bar{A}_k)\big)$ and $\mathrm{tr}(S_b) = \sum_{k=1}^{K} \mathrm{tr}\big((\bar{A}_k - \bar{A})^\top (\bar{A}_k - \bar{A})\big)$.

Let us consider a given sample $s_l$ supposed to belong to group $G_l$. By computing the gradient of $E^2_{\mathrm{LDA}} = \gamma \mathrm{tr}(S_w) - \delta \mathrm{tr}(S_b)$ with respect to $A^{s_l}$, we obtain:

$$
\begin{aligned}
\frac{1}{2}\nabla_{A^{s_l}} E^2_{\mathrm{LDA}} &= \gamma\Big( -\frac{1}{n_l} \sum_{\substack{s \in G_l \\ s \neq s_l}} (A^s - \bar{A}_l) + (1 - \frac{1}{n_l})(A^{s_l} - \bar{A}_l)\Big) \\
&\quad -\delta\Big( \sum_{\substack{k=1 \\ k \neq l}}^{K} -\frac{1}{S}(\bar{A}_k - \bar{A}) + (\frac{1}{n_l} - \frac{1}{S})(\bar{A}_k - \bar{A})\Big)
\end{aligned}
$$

Simplifying the terms and grouping them, we obtain:

$$
\begin{aligned}
\frac{1}{2}\nabla_{A^{s_l}} E^2_{\mathrm{LDA}} &= \gamma A^{s_l} + \frac{\delta}{S}\sum_{k=1}^{K} \bar{A}_k + \frac{\delta}{n_l}\bar{A} \\
&\quad -\gamma \bar{A}_l - \delta \frac{K}{S}\bar{A} - \frac{\delta}{n_l}\bar{A}_l
\end{aligned}
$$

Hence, we obtain the multiplicative update rule given in Eq. 9.

**Alternating constrained least-square** For the alternating constrained least-square update rule, we introduce Lagrange multipliers.

The Lagrangian associated to the problem is defined as follows:

$$
L(\tilde{W}, \lambda) = \mathrm{tr}\big((\mathcal{M} - \tilde{W}\mathcal{R})^\top (\mathcal{M} - \tilde{W}\mathcal{R})\big) + \lambda^\top (\tilde{W}^\top \mathbf{1}_T - \mathbf{1}_P)
$$

where $\mathbf{1}_N$ is a vector of $N$ ones.

Computing the gradient of $L$ with respect to $\tilde{W}$and $\lambda$, we obtain the following system of equations:

$$\begin{aligned} 2\tilde{W}\mathcal{R}\mathcal{R}^\top \quad + \mathbf{1}_P\lambda^\top &= \quad 2\mathcal{M}\mathcal{R}^\top \\ \tilde{W}^\top \mathbf{1}_T \quad &= \quad \mathbf{1}_P \end{aligned}$$

After vectorization using Kronecker products, this can be rewritten:

$$\begin{pmatrix} 2\mathcal{R}\mathcal{R}^\top \otimes I_T & I_P \otimes \mathbf{1}_T \\ I_P \otimes \mathbf{1}_T^\top & 0 \end{pmatrix} \begin{pmatrix} \text{vec}(\tilde{W}) \\ \lambda \end{pmatrix} = \begin{pmatrix} \text{vec}(2\mathcal{M}\mathcal{R}^\top) \\ \mathbf{1}_P \end{pmatrix},$$

which gives the update rule mentioned in the main text. The procedure is similar to obtain the update rule of $W$.
